# Delayed diagnosis of granulomatosis with polyangiitis presenting as irregularly shaped solid lung nodules with heterogeneous enhancement: A case report

**DOI:** 10.1097/MD.0000000000042802

**Published:** 2025-06-06

**Authors:** Ranran Mo, Cuixia Bian, Liping Han

**Affiliations:** a Department of Pulmonary and Critical Care Medicine, Jining No. 1 People’s Hospital, Jining, Shandong, China.

**Keywords:** case report, granulomatosis with polyangiitis, lung nodule, Wegener’s granulomatosis

## Abstract

**Rationale:**

Granulomatosis with polyangiitis (GPA) is a systemic autoimmune vasculitis. Thoracic radiographic findings in GPA can mimic various pulmonary diseases, potentially leading to misdiagnosis. We describe a case of delayed GPA diagnosis, initially presenting as irregularly shaped solid lung nodules with heterogeneous enhancement, such cases are rarely reported.

**Patient concerns:**

A 69-year-old male was initially misdiagnosed with lung cancer based on the identification of solid lung nodules during routine physical examination. Pathological findings from a lung biopsy were inconclusive. Due to the atypical chest computed tomography presentation, the diagnosis was delayed by nearly 2 months and involved 3 different hospitals.

**Diagnosis:**

The definitive clinical diagnosis was GPA complicated by a pulmonary embolism.

**Interventions:**

Methylprednisolone was administered, and immunosuppressive therapy was initiated following infection control.

**Outcomes:**

At outpatient follow-up, most of the patient’s clinical indicators has returned to normal.

**Lessons:**

Pulmonary involvement occurs in over 90% of GPA cases, with characteristic thoracic radiologic findings including solitary or multiple nodules, masses, cavities, and consolidation, with a propensity for lesion recurrence. Clinicians should consider GPA in antibiotic-refractory “pneumonia” with unexplained extrapulmonary manifestations.

## 1. Introduction

Granulomatosis with polyangiitis (GPA), previously termed Wegener’s granulomatosis, is a systemic autoimmune vasculitis characterized by necrotizing granulomas and is strongly associated with anti-neutrophil cytoplasmic antibodies (ANCA).^[1]^ This multisystem disorder predominantly affects the upper and lower respiratory tracts and kidneys, with approximately 90% of cases involving the lungs.^[2]^ Thoracic radiographic findings in GPA can mimic those of various pulmonary diseases, potentially leading to misdiagnosis. Consequently, diagnostic delays exceeding 6 months have been reported in approximately one-third of ANCA-related vasculitis cases.^[3]^ Failure to diagnose can lead to a devastating mortality rate of up to 80% within the first year of disease onset, emphasizing the importance of early identification and management.^[4]^ Here, we describe a unique case of GPA initially presenting as irregularly shaped solid lung nodules with heterogeneous enhancement. Diagnosis was delayed for nearly 2 months and involved 3 different hospitals. The patient provided informed consent for the publication of this case.

## 2. Case presentation

**Figure 1.**
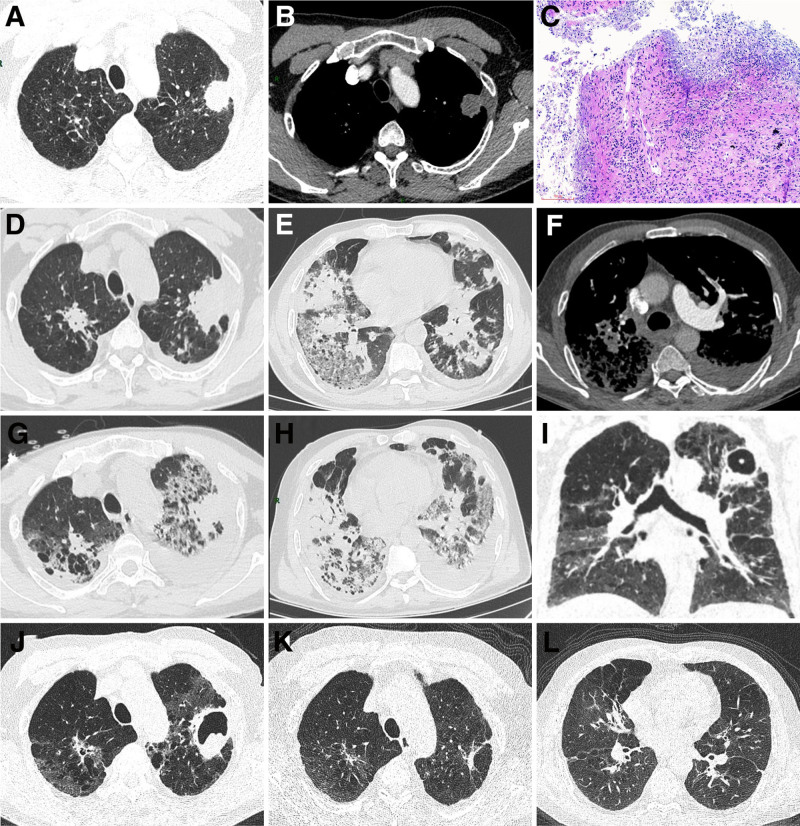
The initial chest enhanced CT scan revealed an irregularly shaped solid nodule with heterogeneous enhancement, which was misdiagnosed as lung cancer (A and B), Pathological HE staining of a puncture of a nodule in the left upper lung, scale bar = 100 µm (C). First CT after admission to our institution (D and E). The computed tomographic pulmonary angiography revealed a thrombus in the left upper pulmonary artery (F). Chest CT findings advances after anti-infective therapy (G and H). Most of the pulmonary lesions were absorbed, leaving behind a circumferential cavity in the upper left lobe 1 months following treatment with methylprednisolone and cyclophosphamide (I and J). The cavity in the upper left lobe becoming obliterated and leaving behind fibrous strands 3 months following treatment (K and L).

A 69-year-old Asian man was admitted to the Department of Thoracic Surgery on June 7, 2022, after the detection of solid lung nodules during a routine physical examination (Fig. 1A and B), with clinical signs suggestive of lung cancer. Cranial magnetic resonance imaging demonstrated sinusitis. A chest computed tomography (CT)-guided percutaneous biopsy of a nodule in the left upper lobe was conducted, in conjunction with thermal ablation. The pathologic analysis demonstrated a granulomatous inflammation with necrosis and multinucleated giant cell reaction, accompanied by focal microabscess formation, and tuberculosis could not be excluded. Special staining results: acid-fast stain (‐), periodic acid-Schiff stain (‐) (Fig. 1C). One week after discharge from the hospital, the patient presented with symptoms of cough, sputum with blood, dyspnea and fever. The maximum body temperature recorded was 38.4 °C, mostly occurring in the late afternoon and at night. The patient was admitted to the Hospital for Infectious Diseases on June 26, 2022. He was clinically diagnosed with “pulmonary tuberculosis” and received antituberculosis treatment for 23 days. While hospitalized the patient developed a rash on both lower extremities. These rashes resolved after treatment with methylprednisolone and loratadine. However, the patient’s symptoms persisted, and thoracic CT scan demonstrated radiological progression. Consequently, the patient was transferred to our institution on July 19, 2022.

Upon admission, a thorough physical examination revealed diminished respiratory sounds in the left lower lobe and moist rales in both lungs. Review of the thoracic CT scans revealed multiple consolidations and nodules in both lungs, with worsening compared to the previous scan conducted on June 11, 2022 (Fig. 1D and E). Blood gas analysis indicated type I respiratory failure, with a partial pressure of oxygen of 49 mm Hg and an oxygenation index of 233 mm Hg. A combination of imipenem/cilastatin and trimethoprim-sulfamethoxazole was administered. During treatment, although the fever subsided, the volume of hemoptysis increased compared to pretreatment levels. The patient’s dyspnea worsened, and his oxygenation index decreased to 122 mm Hg. D-dimer levels increased to 20.60 mg/L. Subsequent The computed tomographic pulmonary angiography revealed a thrombus in the left upper pulmonary artery, consistent with pulmonary embolism (Fig. 1F). Bilateral lower extremity venous ultrasonography revealed thrombosis in the intermuscular veins on the dorsal aspect of both calves. The patient was newly diagnosed with pulmonary embolism; however, due to the co-occurrence of hemoptysis, anticoagulation therapy was not initiated. Despite adjustments to the treatment regimen, there was no improvement in the patient’s symptoms or radiographic findings (Fig. 1G and H). Laboratory tests for rheumatic indicators were conducted, and proteinase 3 (PR3)-ANCA was positive, with a level exceeding 200 RU/mL: well above the reference range of 0 to 20 RU/mL. According to the 2022 classification criteria for GPA, the patient scored 10 points, unequivocally confirming the diagnosis of GPA.^[5]^ The definitive diagnosis was GPA complicated by pulmonary embolism. Methylprednisolone at 1 mg/kg/day was administered, and immunosuppressive therapy was initiated following infection control. Oral methylprednisolone and cyclophosphamide were administered upon discharge. One month after discharge, the patient reported significant relief from dyspnea, with decreased sputum and reduced hemoptysis. Thoracic CT showed that most of the pulmonary lesions had been absorbed, leaving behind a cavity in the upper left lobe (Fig. 1I and J). Three months later, the PR3-ANCA level had normalized, and thoracic CT scan revealed resolution of the cavity in the upper left lobe, leaving behind fibrous strands (Fig. 1K and L).

## 3. Discussion

GPA is an uncommon small-to-medium vessel necrotizing vasculitis that can involve nearly any organ, presenting with a broad clinical spectrum ranging from localized disease to severe, life-threatening forms.^[6]^ The classic clinical triad consists of upper and lower respiratory tract and renal disease. Other less frequently involved organ systems include the skin, musculoskeletal system, ocular, cardiovascular, and peripheral nervous system. Constitutional symptoms such as weight loss, fever, fatigue, and malaise are commonly present.^[7]^ Elevation of serum PR3-ANCA titers occurs in most patients with clinical diagnoses of active GPA, but a significant minority are myeloperoxidase-ANCA positive or have a negative ANCA.^[8]^ Granulomatous inflammation and multinucleated giant cells are key definitive pathological hallmarks in the diagnostic evaluation of GPA.^[6,9]^ The diagnosis of GPA is based on a combination of clinical evaluations, imaging findings, laboratory tests, and histopathologic results.

Pulmonary involvement occurs in more than 90% of GPA cases during the disease course, and the diversity of thoracic radiological findings reflects the variability of underlying pathological changes.^[2]^ When GPA is combined with an infection, it becomes more challenging to distinguish it from tuberculosis, lung cancer, or other pulmonary diseases. Characteristic thoracic CT features of GPA include solitary or multiple nodules, masses, cavities, and consolidation, with a propensity for lesion recurrence.^[10]^ The most common CT finding is multiple, varying-sized, round-to-oval nodules or masses, often exhibiting spiculation and fibrous strands extending to the adjacent pleural surfaces. These nodules preferentially localize to the peripheral zones of the middle and lower lung fields, as well as the subpleural regions. On contrast-enhanced CT, peripheral enhancement is often observed, while central necrosis appears non-enhancing; the “feeding vessel sign” may also be observed.^[10,11]^ In this case, the initial CT scan showed multiple round nodules in the peripheral and subpleural regions of both lungs, with spiculation and pleural adhesions. Contrast-enhanced scans revealed heterogeneous enhancement, consistent with characteristic features of GPA (Fig. 1A and B). Central cavitation occurs in up to 50% of cases and results from necrosis of the lung nodules.^[12]^ Cavities can take various forms, including thick-walled, thin-walled, and circumferential structures. Circumferential cavities are the most characteristic and defined by irregular inner edges devoid of calcification.^[13]^ After treatment, the nodules in both lungs were partially absorbed, while the nodule in the left upper lobe evolved into a ring-shaped cavity containing a central nodule, manifesting as the classic “island sign” (Fig. 1I and J). Reports of GPA coexisting with venous thromboembolism remain relatively rare. We hypothesize that these mechanisms may be related to vascular endothelial injury and a hypercoagulable state induced by systemic vasculitis.^[14]^

This case highlights the complexity of GPA, its diverse radiological presentations, and the importance of a holistic diagnostic approach. We summarized the patient’s clinical course and propose several Take-Home Messages to guide the management of similar cases:

- GPA is a rare necrotizing necrotizing small-to-medium vessel vasculitis that can affect nearly any organ. The classic clinical triad involves the upper and lower respiratory tract and the kidney.
- Pulmonary involvement occurs in over 90% of GPA cases, with characteristic thoracic radiologic findings including solitary or multiple nodules, masses, cavities, and consolidation, with a propensity for lesion recurrence.
- Elevation of PR3-ANCA titers usually occurs in most GPA.
- Adopt time-bound reassessment (e.g., 2-week intervals) to avoid delayed diagnosis in atypical presentations of GPA.
- Clinicians should consider GPA in antibiotic-refractory “pneumonia” with unexplained extrapulmonary manifestations (e.g., sinusitis, hematuria).

## Acknowledgments

We sincerely thank the patient for providing informed consent to participate in this case report and for allowing the publication of this case report.

## Author contributions

**Investigation:** Ranran Mo.

**Methodology:** Ranran Mo.

**Supervision:** Cuixia Bian, Liping Han.

**Validation:** Cuixia Bian.

**Writing – original draft:** Ranran Mo.

**Writing – review & editing:** Liping Han.
